# Spontaneous emergence of computation in network cascades

**DOI:** 10.1038/s41598-022-19218-0

**Published:** 2022-09-02

**Authors:** Galen Wilkerson, Sotiris Moschoyiannis, Henrik Jeldtoft Jensen

**Affiliations:** 1 School of Computer Science and Electronic Engineering, University of Surrey, Guildford, GU2 7XH UK; 2 Department of Mathematics, Centre for Complexity Science, Imperial College London, South Kensington Campus, London, SW7 2AZ UK; 3 Institute of Innovative Research, Tokyo Institute of Technology, 4259, Nagatsuta-cho, Yokohama, 226-8502 Japan

**Keywords:** Cellular signalling networks, Computational models, Computational neuroscience, Learning algorithms, Network models, Applied mathematics, Computational science, Computer science, Pure mathematics, Statistics, Complex networks, Nonlinear phenomena, Phase transitions and critical phenomena, Statistical physics, Phase transitions and critical phenomena, Quantum information, Qubits, Biological physics, Electronics, photonics and device physics, Information theory and computation, Statistical physics, thermodynamics and nonlinear dynamics, Computational neuroscience, Neural circuits, Computational biology and bioinformatics, Neuroscience, Mathematics and computing, Physics

## Abstract

Neuronal network computation and computation by avalanche supporting networks are of interest to the fields of physics, computer science (computation theory as well as statistical or machine learning) and neuroscience. Here we show that computation of complex Boolean functions arises spontaneously in threshold networks as a function of connectivity and antagonism (inhibition), computed by *logic automata (motifs)* in the form of *computational cascades*. We explain the emergent inverse relationship between the computational complexity of the motifs and their rank-ordering by function probabilities due to motifs, and its relationship to symmetry in function space. We also show that the optimal fraction of inhibition observed here supports results in computational neuroscience, relating to optimal information processing.

## Introduction

The relationship between physical systems and information has been of increasing and compelling interest in the domains of physics^1–3^, neuroscience^4,5^, computer science^6–12^, quantum computing^2,13,14^, and other fields such as computation in social networks^15–17^, or biology^18,19^ to the point where some consider information to be a fundamental phenomenon in the universe^20–22^. Often, physical systems operating on information take place on, or can be modeled by, network activity^23^, since information is transmitted and processed by interactions between physical entities.

The principle of Occam’s razor and goals of achieving a deeper understanding of these physical-information interactions encourage us to find the simplest possible processes achieving computation. Thus we may conduct *basic research into understanding necessary and sufficient conditions for systems to perform information processing*. Cascades, particularly on networks, are such a simple and ubiquitous process. Cascades are found in a great number of systems-the brain, social networks, chemical-, physical-, and biological-systems-occurring as neuronal avalanches, information diffusion, influence spreading, chemical reactions, chain reactions, activity in granular media, forest fires or metabolic activity, to name a few^23–28^. The Linear Threshold Model (LTM) is among the simplest theoretical models to undergo cascades. As a simple threshold network, the LTM is also similar to artificial models of neural networks, without topology restrictions such as layering, or edge weights^12^.

Since the work of Shannon^29^, the *bit* has been considered the basic unit of information. Therefore, whatever we can learn about processing of bits can be extended to information processing in non-Boolean systems. The tools of Boolean logic then allow us to begin to develop a formalism linking LTM and other cascades to information processing in the theory of computing^30^. In systems of computation or statistical learning, patterns of inputs are mapped to patterns of output by Boolean functions^12,31^.Another way to express this is that a bit is the simplest possible perturbation of a system. Bits can interact via some medium, these interactions can be represented by edges in a network, and Boolean functions describe the results of possible interaction patterns.

Since we aim to study this topic from first principles, we are interested in how the combinatorial space of possible networks interacts with the combinatorial space of possible Boolean functions, via cascades and the control parameters. Particularly, we would like to understand the *phase space of Boolean functions* computed by LTM nodes on the input (seed) nodes by the cascade action.

Two of the current authors have previously published a work on Boolean functions computed by the Linear Threshold Model^32^. In that work, the focus was to show universal computing, sources of efficiency, including geometric constraints, and one way to consider the LTM as a statistical classifier that can be trained. Here, as stated, we simply aim to understand the phase space of the logic functions computed by the LTM with respect to network connectivity. The focus here is not on dynamics or learning in these networks.

From a mathematical perspective, we can treat the brain or other natural systems having *N* elements in the worst case as a random network, where there are $N(N-1)/2$ possible connections, yielding $2^{(N(N-1)/2)}$ possible networks. Meanwhile, the space of Boolean functions grows exceptionally quickly. There are $2^{2^k}$ unique Boolean functions on *k* inputs. This immediately makes us ask how this space behaves, and how large networks such as the brain can navigate toward particular functions in this vast space. We also observe that for the all the functions available on *k* inputs, the decision tree complexity (depth of decision tree computing them) appears exponentially distributed, meaning that the vast majority of functions available are complex as *k* increases.

A somewhat surprising initial result in this investigation is that *complex functions on inputs emerge spontaneously and seemingly inevitably as threshold networks are connected at random*.

## Linear threshold model (LTM), Boolean logic, and antagonism

The Linear Threshold model (LTM)^23^ is defined as follows: A random (Erdos-Renyi-Gilbert) undirected graph is constructed, having *N* nodes and *p*, the probability of an edge between each pair of nodes. Each node is then assigned a random threshold $\phi$ from a uniform distribution, $\phi \sim U[0,1]$. Nodes can be *unlabelled* or *labelled*, corresponding to 0 or 1 respectively, and are all initialized as *unlabelled*. To run the cascade, a small set of input seed nodes are *perturbed*, marked as labelled. Now, each unlabelled node *u* is examined randomly and asynchronously, and the fraction of its graph neighbors that are labelled $\big (\frac{L(u)}{deg(u)}\big )$ is determined, where *L*(*u*) is the number of *u*’s neighbors that are labeled, and *deg*(*u*) is *u*’s degree. If *u*’s fraction reaches its threshold $\big (\frac{L(u)}{deg(u)}\ge \phi \big )$, *u* is marked labelled. This process continues until no more nodes become labelled. Here we note that the LTM may be written in vector form, and bears some similarity to the artificial McCulloch–Pitts neuron^12^. It has been shown that the LTM exhibits *percolation*, where a giant connected component (GCC) of easily-influenced *vulnerable* nodes *u* (having $\phi \le 1/deg(u)$, suddenly arises at the critical connectivity^23^.

We observe that cascades in the LTM compute monotone Boolean functions (the number of true outputs cannot decrease in the number of true inputs) at each non-seed node on the input perturbation patterns^33^. In our numerical experiments, we create the LTM as above, but choose input seed nodes *a* and *b* (for $k = 2$ inputs) as the only possible loci of initial perturbation. In one trial, we create a network, freezing network edges and thresholds across all possible input patterns (Table 1, cols. a, b). For each input pattern we reset non-seed nodes to unlabelled, set seeds according to inputs, and run the cascade. We then identify the function computed by each non-seed node ($f_0, \ldots , f_{15}$) (Table 1, cols. 0–15). Functions are numbered according to the integer value of their bit string.

**Table 1:** Truth tables for binary functions. The truth tables of all possible unique binary $(k =2)$ Boolean functions are shown ($2^{2^k} = 16$ functions).

Inputs		Functions															
a	b	0	1	2	3	4	5	6	7	8	9	10	11	12	13	14	15
0	0	0	0	0	0	0	0	0	0	1	1	1	1	1	1	1	1
0	1	0	0	0	0	1	1	1	1	0	0	0	0	1	1	1	1
1	0	0	0	1	1	0	0	1	1	0	0	1	1	0	0	1	1
1	1	0	1	0	1	0	1	0	1	0	1	0	1	0	1	0	1

The LTM can only compute *monotonically-increasing* Boolean functions (columns 0, 1, 3, 5, 7), where the first row equals zero, since the seed nodes are unlabelled (here 0 and 1 correspond to unlabelled or labelled, respectively). Thus, it cannot compute functions 2, 4, 6, or 8 to 15.

**Figure 1:**
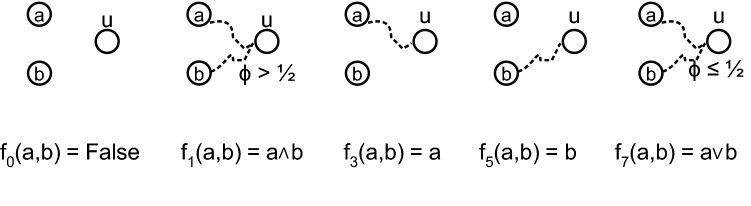
*Logic motifs* compute Boolean functions. The simplest LTM sub-networks are logical automata *(logic motifs)* and compute the monotone functions for $k = 2$ inputs at node *u* on perturbations of *a* and *b*. Dashed lines are network paths.

The zero function, $f_0(a,b) = 0$ (False) is computed by a simple sub-network, where node *u* has no path to either seed node (Fig. 1). Similarly, function $f_1(a,b) = a \wedge b$ (AND) is computed by *u* with a sub-network having paths from both seed nodes *a*, *b*, and a threshold $\phi > \frac{1}{2}$. Similar sub-networks allow us to obtain nodes computing monotone functions $f_3, f_5, f_7$ (Fig. 1). These sub-networks are therefore logical automata^30,34^, and we note that they form functional *logic motifs* in the network^35,36^.

**Figure 2:**
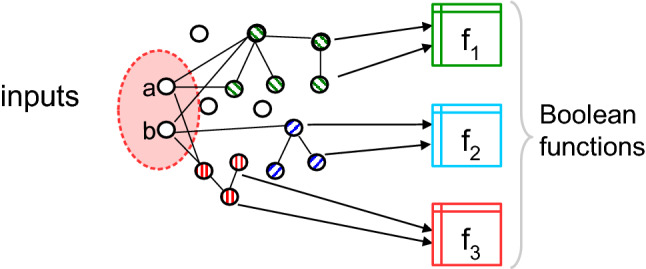
LTM nodes compute Boolean functions in *computational cascades*. Iterating through all possible perturbations of input seed nodes *a* and *b*, each non-seed network node must compute some Boolean function on the inputs.

We find that an LTM network cascade will yield a distribution of Boolean functions on its input nodes, and the possible functions computed by non-seed network nodes will partition the set of monotone Boolean functions (Fig. 2) (with the exception of $f_{15}$). Thus the LTM carries out *computational cascades* on input perturbation patterns.

We then obtain monotonically decreasing functions (negation of the LTM), by taking the logical complement of the original LTM labelling rule, so that some node *u* is instead activated when its *fraction of labelled neighbors is less than its threshold* $\big (\frac{L(u)}{deg(u)} < \phi \big )$. We call such nodes *antagonistic*, from which we can construct an *antagonistic linear threshold model (ALTM)*. For 2 inputs, replacing *u* with an ALTM node $\lnot u$, will compute $f_{15}, f_{14}, f_{12}, f_{10}$, and $f_8$ (Table 1), and the sub-networks are antagonistic versions of those for $f_0, f_1, f_3, f_5,$ and $f_7$, respectively (Fig. 1).

**Figure 3:**
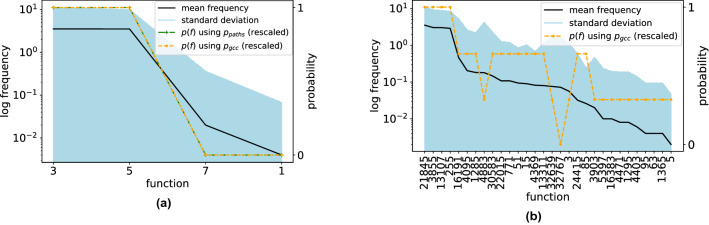
Function frequency corresponds to probability of required paths in a rank-ordering. (**a**) Logarithmic frequency of non-zero functions computed by the ensemble of LTM cascades for $N = 10000$ nodes, average degree $z = 4$ and $k = 2$ inputs, over 500 realizations reveals an apparent rank-ordering (solid line). Mean frequency is proportional to path probabilities, having a Pearson correlation of 1.0, both predicted by probabilities derived from logic motifs using $p_{path}$ (‘+’) [e.g. see Eq. (1)], and complexity $p_{gcc}^{C(f) + 1}$ (large dot) Eq. (4) (rescaled, overlaid, both dashed). Thus Eq. (4) also well-predicts Eq. (1). Frequency therefore varies inversely with decision tree complexity *C* (‘+’). (**b**) Rank-ordering is more evident for $k = 4$ inputs, appearing as a decreasing exponential with goodness of fit $r^2 = 0.88$. Here, Pearson correlation between *p*(*f*) and mean frequency is 0.74. Shaded regions are one standard deviation. Probabilities have been centered and normalized.

A sufficiently large ALTM, by composing monotone decreasing functions (e.g. NAND, NOR), can undergo a cascade to compute any logical function on its nodes, forming a *universal basis*^31^.

## Statistics of attractors in the Boolean function space

We experiment first on the LTM, to investigate the observed frequency of Boolean functions in simulation. We created networks having $N = 10000$ nodes and $k = 2$ inputs, in a range of mean degree $z = [0, \frac{1}{2}, 1, 2, 4, 8, 16, 64, 256, 1024, 4096, 10000]$, over 10000 trials for each *z* value. At mean degree $z = 4$, shortly after emergence of the GCC, we observed that the frequency of functions is very skewed (Fig. 3a). Experiments for $k = 4$ inputs, again for $N = 10000$ nodes at mean degree $z = 4$, ensembled over 500 realizations, also yield an approximate exponential decay of the rank ordering function (Fig. 3b). (See Supplemental Information A.4.)

We investigate the skewed distribution of these functions by asking *“What is the probability of obtaining the simplest network to compute each of these functions?”*. From (Fig. 1), we can derive the probability of each monotone function. For example, if there is no path from seed nodes *a* and *b* to some node *u* we obtain $f_0$, thus

$$\begin{aligned} p(f_0) \propto (1 - p_{path})^2, \end{aligned}$$

where $p_{path}$ is the probability of a path between two randomly chosen nodes.

The function $f_1$ requires paths from *a* and *b* to *u*, thus

1 $$\begin{aligned} p(f_1) \propto p_{path}^2. \end{aligned}$$

However, with percolation in mind, we observe that for large graphs, the probability of paths between *n* nodes approaches the probability that all *n* nodes belong to the giant connnected component (GCC)^25,37^.

This gives us, again from (Fig. 1),

$$\begin{aligned} p(f_1) \propto p_{path(a,b,u)} \propto p_{gcc}^3, \end{aligned}$$

where $p_{gcc}$ is the probability for a random node to belong to the GCC.

Let’s define $v = P_{gcc}$. From^37^, we know that *v* is given implicitly by the following relation

2 $$\begin{aligned} v = 1 - e^{-zv}, \end{aligned}$$

where *z* is the mean degree. (See Supplemental Information A.3 for more information.)

We subsequently observe that the number of required paths from seed nodes to node *u*, which computes monotone function *f*, is equal to the *decision tree complexity *(*C*), the depth of the shortest decision tree to compute *f*. In order for *u* to decide the value of a seed node, the seed’s perturbation information must be transmitted along a path to *u*.

Taking a Boolean function’s Hamming cube representation, its decision tree complexity *C* is complementary to the number of congruent axial reflections *R* along each of its axes *D* (details in Supplemental Information A.1). That is, if a Boolean function’s Hamming cube is constant along an axis, it is independent of that axis, giving us

3 $$\begin{aligned} C = D - R. \end{aligned}$$

In other words, *the number of paths a monotone Boolean function requires is exactly the number of axial reflection asymmetries of its Hamming cube.* This allows us to relate a function’s symmetry to decision tree complexity and then to its frequency. Recall that the critical percolation threshold in an arbitrarily large Erdos-Renyi-Gilbert graph occurs at critical mean degree $z_c = 1$, a very small connectivity. Since $p_c = \frac{z_c}{N-1}$, critical connection probability $p_c \ll 1$. Therefore, the network will contain a GCC and remain tree-like even for substantial mean degree above $z_c = 1$, since the clustering coefficient $C_{\mathrm{clus}} \approx p$^25^. (E.g. for $N =10000$ and $z = 10$, $C_{clus} \approx 1/1000$.) In a tree, the number of nodes is one more than the number of edges $N = |E| + 1$. Thus, as $p \rightarrow p_c$,

4 $$\begin{aligned} p(f) \propto p_{gcc}^{C(f) + 1}. \end{aligned}$$

Indeed it appears that Eq. (4) is highly correlated with the probabilities derived from logic motifs Eq. (1), and that observed function frequency is proportional to Eq. (4) as well (Fig. 3a), having a Pearson correlation of approximately 1.0 for k = 2, and 0.74 for k = 4. This also shows, due to Eq. (4) an inverse rank ordering relation between frequency and decision-tree complexity, appearing as a decreasing exponential in frequency. Given that, as mentioned in the introduction, there is an increasing exponential distribution of decision tree complexity in the truth table of all Boolean functions, this result is especially surprising.

### Function distribution with antagonism

**Figure 4:**
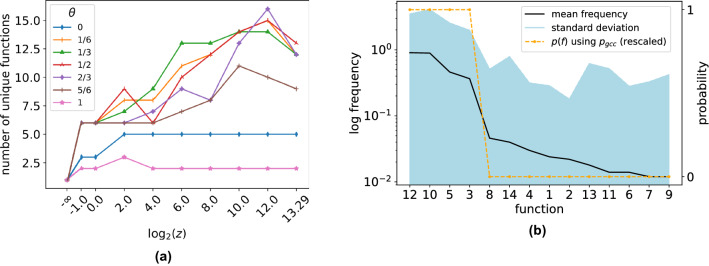
Antagonism fraction ($\theta$) agrees with biology; non-monotone functions also predicted by path requirements. (**a**) For networks with $N = 10000$ nodes and $k = 2$ inputs, over 500 realizations, varying the mean degree *z* and fraction of antagonistic nodes $\theta \in \{0, \frac{1}{6}, \frac{2}{6},\ldots 1\}$, we observe that the mean number of unique functions per network is maximized over several orders of magnitude ($z \in [2^3, 2^{10}]$) by networks having a fraction of antagonistic nodes $\theta = \frac{1}{3}$ (triangles), coinciding with other findings^38^. (**b**) At $\theta = \frac{1}{3}$ and $z = 2^6$, we again observe a skewed frequency, and a proportional relationship between function frequency and probability due to complexity Eq. (4), having Pearson correlation of 0.91. Shaded region is one standard deviation. Probabilities have been centered and normalized. (Functions $f_0$ and $f_{15}$ have been removed, since in the ALTM they can occur outside of the GCC).

A similar simulation, having $N = 10000$ nodes, $k = 2$ inputs, ensembled over 500 trials in a range of mean degree values *z* and fraction of antagonistic nodes $\theta \in \{0, \frac{1}{6}, \frac{2}{6},... 1\}$, reveals a sudden increase in the number of unique non-zero functions vs. both *z* and $\theta$ (Fig. 4a). The number of unique functions is maximized over several orders of magnitude near criticality, for $z \in [2^3, 2^{10}$], and $\theta = 1/3$. Observing that antagonism and inhibition are interchangeable^12^ (Supplemental Information A.2) , this lends support to optimal information processing around $30 \%$ inhibition, found in other research^38^, and why this fraction of inhibitory neurons seems prevalent biologically.

**Figure 5:**
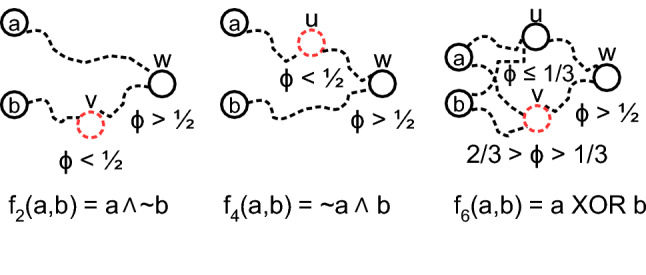
Motifs for non-monotone functions. Simplest *logic motifs* to compute non-monotone Boolean functions $\{f_2$, $f_4$, $f_6\}$ (Table 1) in the ALTM at random node *u*, on seed nodes *a*, *b*. Dashed lines represent paths, and dashed nodes are antagonistic. Functions $f_{13}$, $f_{11}$, and $f_9$ are negations of these, respectively, so have very similar networks, negating each node.

For this mix of LTM and ALTM nodes, we again observe a similar rank-ordering of functions, here at $z = 64, \theta = 1/3$, and that, as in the LTM, frequency is again proportional to probability derived from function complexity (Fig. 4b), having a Pearson correlation of 0.91.

We note, however, that Eq. (3) under-estimates the number of paths required for non-monotone functions. For example, $f_6$ (XOR) requires 6 paths between 5 nodes, all of which must be in the GCC (Fig. 5), so that $p(f_6) \propto p_{gcc}^5$. However, this function’s decision tree complexity $C = 2$, predicting by Eq. (4) that $p(f_6) \propto p_{gcc}^3$. Therefore a more informative complexity measure is needed for non-monotone functions.

## Discussion

As indicated in the title, we see the main result of interest as the spontaneous emergence of complex logic functions in the minimally-constrained random threshold networks. This then implies that many physical, biological, or other systems are able to perform such computation by ubiquitous avalanches or cascades.

We note that this result also begins to give us an explanation of the *criticality hypothesis* vis-à-vis neuroscience^39–41^. That is, at the critical threshold, with the emergence of the giant component, the number of unique functions spontaneously increases. Along with that comes an increase in the number of complex functions. As neuronal networks need to compute integrative complex functions on sensory information, or on information passed between modular areas in the brain, the utility of this complexity is self-evident^4^. We note that in computational neuroscience, there is also discussion of the integration of information and complexity or consciousness^42,43^. These motifs therefore give us a starting point for the relationship between structure and function as well.

Also, the present work connects to machine- or statistical-learning, where in classification, Boolean functions are computed on high-dimensional data. Until now, however, despite their ubiquity in nature, neither criticality nor cascades have played a large role in machine learning as a design paradigm or analytical framework^12^. We see this as a large potential opportunity to improve deep learning methods.

The spontaneous emergence of complex computation is an example of a symmetry breaking phase transition, as the giant connected component (spanning cluster) comes into existence at the critical connectivity^44,45^. We conjecture that we are witnessing how *complexity of functionality results from symmetry breaking in systems*^45^. This complexity takes on a distribution that reflects a hierarchy in an exponential rank-ordering law.

We also see that, from a larger theoretical perspective, the confluence of cascades (percolation branching processes) and information processing by Boolean logic stands at the intersection between several very large and highly developed areas of research—percolation- and computational automata-theory^27,30^.

The specific mechanism of the logical automata realized by *logic motifs* extends previous work about network motifs and their function, mainly in the genetic domain^35^, into many other areas, again due to the ubiquity of cascades in threshold networks. The observance of logic motifs as automata also allows us to change our perspective on network percolation. In the past, we saw it perhaps only in terms of connected component size distribution. Now, however, we may view these components as a *zoo or library of functions*, available to the network by connection, much as importing a function occurs in programming languages. We note that the scale invariance at criticality may exist at the Pareto-optimal point between complexity and diversity. That is, there will be a small number of larger components computing complex functions, and a great number of very small, simple components having a large variety of thresholds.

### Future work

In developing this work, we inevitably stumbled across an overwhelming number of ideas and directions that we can take. We can only briefly list them.

We have seen above that other complexity measures could be found for non-monotone functions, to better predict their frequency in mixed LTM/ALTM networks. We suspect that Boolean Fourier analysis would be fruitful here. We also expect that, for larger inputs, these non-monotone functions will dominate the function space, and that the Hamming cube symmetries make it possible to write a partition function for them. Along with this, it should be possible to predict more exact probabilities of functions, which depend on the occurrence of cascades being blocked, and of nodes inheriting their neighbors’ complexity, among other factors.

We would also like to generalize these predictions to $k \gg 2$ inputs and much larger networks ($N \sim 10^9$ nodes), while understanding mechanisms and heuristics for learning by studying re-wiring dynamics and self-organized criticality^46^ in these large combinatorial spaces. For example, we suspect that modularity develops as a network’s capacity to extract complexity from inputs is exhausted. We also suspect that function distribution can be understood in terms of multiple network density percolation thresholds, depending on function path requirements, more evident for larger inputs.

Furthermore, we intend to study the relation between function and network symmetry in the context of symmetry breaking. We conjecture, for example, that there is a conservation law of complexity or information, meaning that what we call computation comes at the expense of lost information, rendering the network a kind of *information engine*^47^, whose output is *computation*, and that this lies at the heart of information creation.

Of course, it could also be fruitful to understand this work in terms of information processing, using measures such as transfer entropy, of increasing use in computational neuroscience and automata theory^11^. Along with this we see an opportunity to formalize the *criticality hypothesis* in light of our results on computation. In the hypothesis, avalanche criticality (the kind of percolation seen here) and so-called *edge of chaos* are convolved qualitatively, by saying that information processing is optimized ‘near criticality’^48,49^.

We would like to research the effects of geographic energy constraints and other network topologies, found in real-world systems, on the function phase space. For example we conjecture that both modularity and layering will result from restricting geographic connection distance, with a result that complex functions appear at nodes on the surface (or interface) of networks, convenient for passing to subsequent networks.

Finally, although we have used the term *computation* here, it would be useful to carefully study the linear threshold model as a computing machine, especially when re-wiring, investigating its Turing completeness, run-time, and related phenomena.

## Conclusion

Here we have shown that the Linear Threshold Model computes a distribution of monotone Boolean logic functions on perturbation inputs at each node in its network, and that with the introduction of antagonism (inhibition), any function can be computed. Notably, complex functions arise in an apparent exponentially decreasing rank-ordering due to their requirements for perturbation information from seed nodes, and these requirements correspond to their functional asymmetries. These asymmetries can be used to obtain their probability exponent as a function of the probability of belonging to the network’s giant connected component. Finally, we observe that the number of unique functions computed by an LTM of mixed excitatory and antagonistic nodes is maximized near 1/3 antagonism, over several orders of magnitude of connectivity, coinciding with other research.

## Supplementary Information

Supplementary Information.

## Data Availability

Data from simulations is available here: https://github.com/galenwilkerson/Spontaneous-Emergence-of-Computation-in-Network-Cascades Data is in numpy array format, saved as python .pkl files, then gzipped.
