# Supplementary material for: Spontaneous emergence of computation in network cascades
Source: Sci Rep. 2022 Sep 2;12:14925. doi: 10.1038/s41598-022-19218-0 (PMC9440044; doi:10.1038/s41598-022-19218-0)
Supplement: Supplementary file 1 — Supplementary Information. [file 41598_2022_19218_MOESM1_ESM.pdf]

A Supplemental Information

A.1 Algorithm for determining Decision Tree Complexity

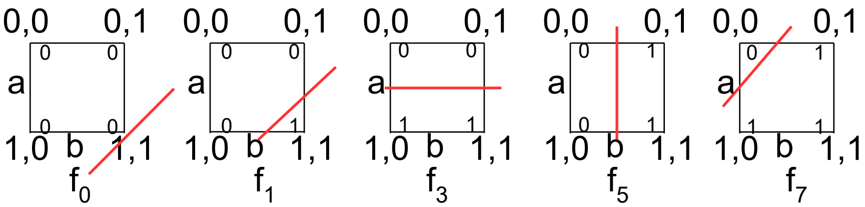

Figure 1. Hamming cube representations of LTM-computable monotone Boolean functions of two variables. Line represents linear separator. Note that for monotone functions, true values must be below or to the right of false values.

Algorithm 1 Decision Tree Complexity using Hamming Cube reflections

---

$H \leftarrow (x, f(x))$ ▷ create labelled Hamming cube from inputs, outputs
$R \leftarrow 0$ ▷ number of congruent reflections
for $d \in D$ **do** ▷ for each dimension
  $\Delta r = \begin{cases} 1 & (H \equiv (H_d)) \\ 0 & otherwise \end{cases}$
  $R \leftarrow R + \Delta r$
$C \leftarrow D - R$ ▷ Complexity = dimensionality - congruent reflections

---

To determine a Boolean function's Decision Tree Complexity, we create the Hamming cube H on the input values of the function. The number of axes D of H is equal to the number of inputs (k). We then label each corner of H according to the function values f [Fig. 1].

For each input variable d of the cube's D axes, we reflect the Hamming cube about that axis, obtaining the reflected Hamming cube H_d. If $H \equiv H_d$, we add one to the reflection symmetry R.

The Decision Tree Complexity C is then the dimensionality D minus the number of congruent reflections R ($C = D - R$). The intuition is that if the Hamming cube of a particular function is congruent to an axial reflection, the function is independent of that axis.

Thus, paths and their resulting cascades break symmetry and create complexity in the network, realized in the function order parameters.

A.2 Interchangeability of Antagonism and Inhibition

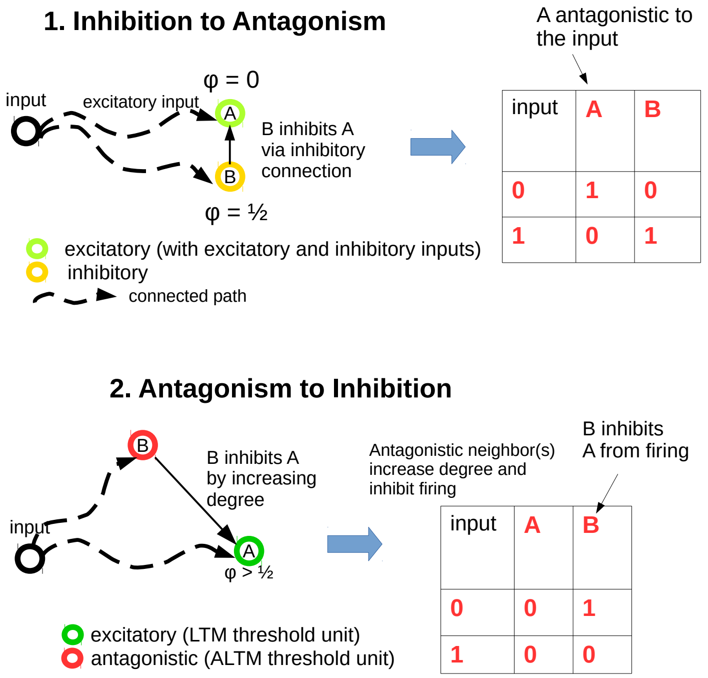

Figure 2. Simplest sub-networks to convert between inhibition and antagonism. Both sub-networks have 2 internal nodes, which implies that there is a 1:1 ratio in the minimal number of nodes to perform either operation. (Top) If A has inhibitory input from node B, whose activation can prevent A's activation, then the truth table shows that A is antagonistic to the input. (Bottom) By increasing node A's degree, antagonistic node B inhibits the activation of node A when input is activated.

A.3 Probability of a node belonging to the Giant Connected Component

From[1–3], we have the derivation of the probability of a random node in an Erdos-Renyi graph to be in the giant connected component (GCC):

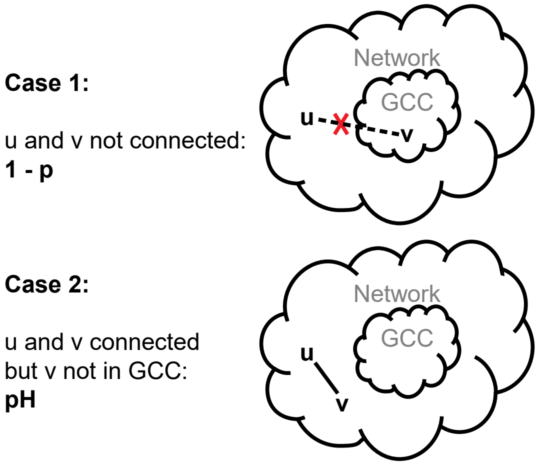

Figure 3. Derivation of p_{gcc}. Two ways that a vertex u can not be in the GCC.

Let H be the probability that some randomly chosen vertex is not in the giant connected component (GCC),

$H = prob(v \notin GCC)$.

Let u be some node not in the spanning cluster. For every other node v in the network, there are 2 cases:

1. u is not connected to v,

   $1 - p$.

2. u is connected to v and v is not in the GCC,

   pH.

There are $n-1$ vertices (u) to check, hence

$H = ((1-p) + pH)^{n-1}$

Since
$p \approx \frac{z}{n}, p > 0$.

This gives us

$H = (1 - \frac{z}{n} + (\frac{z}{n})H)^{n-1}$

$H = (1 - \frac{z}{n}(1-H))^{n-1}$

$\log H = \log(1 - \frac{z}{n}(1-H))^{n-1}$

$\log H = (n-1)\log(1 - \frac{z}{n}(1-H))$

$\log H \approx (n-1)(1-\frac{z}{n}(1-H))$

(Using the fact that $\log(1+x) \approx x$ for small x.)

$\log H \approx (n-1)(-\frac{z}{n}(1-H))$

$\log H \approx -\frac{z(n-1)}{n}(1-H)$

$\log H \approx -z(1-H)$

$H \approx e^{-z(1-H)}$,

Finally, for the frequency of the vertices in the giant component $v = 1-H$ we obtain
$1-v = e^{-zv}$.

or

$p(u \in GCC) = v = 1-e^{-zv}$.

This can be solved numerically, and plotted graphically [Fig. 4, left].

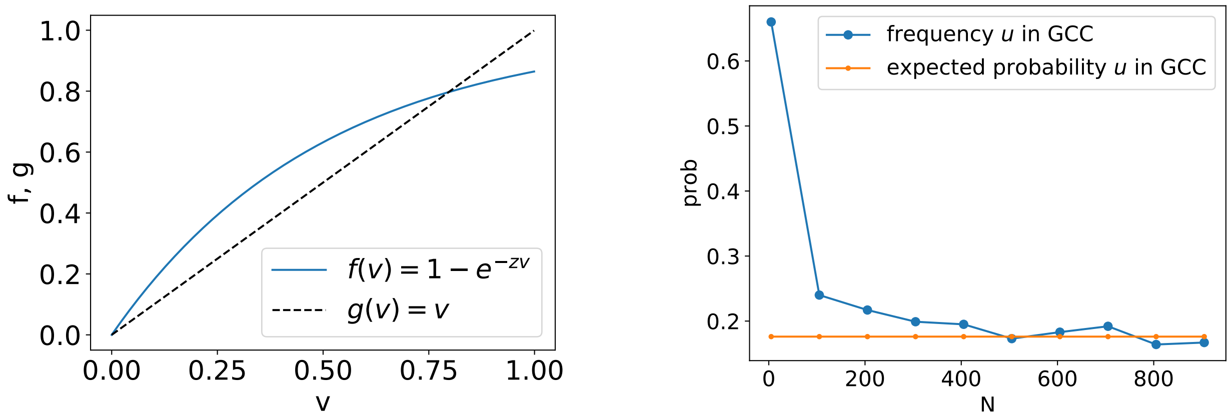

Figure 4. Solutions of p_{gcc}; (Left) The probability $p_{gcc} = v$ for a randomly chosen node to belong to the GCC has a non-zero solution when mean degree $z > 1$[2]. Here $z = 2$. (Right) Observed frequency in GCC converges to prediction as $N \to \infty$.

We observe that for networks above 800 nodes, the frequency of a random node being in the GCC approaches the predicted probability as the number of nodes $N \to \infty$ [Fig. 4, right].

This can be tested by comparing the above closed form probability to the probability of there being a path between several nodes. Here we have tested the path frequency between three nodes vs. mean degree z against the closed form prediction [Fig. 5]. This is generally very well predicted, falling within the 90% confidence interval.

The establishment of the giant component is important for the cascade, here we remind the reader how it behaves [Fig. 6], as the probability of a random node to belong in the GCC suddenly increases at mean degree $z = 1$.

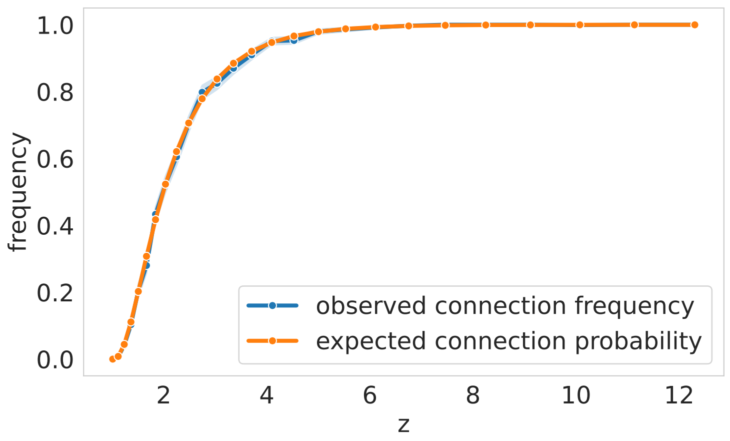

Figure 5. Solutions of p_{gcc}; p_{gcc} well-predicts observed path frequency. The probability of 3 nodes belonging to the GCC in an Erdos-Renyi-Gilbert network well-estimates the observed frequency of a path between them (N = 10000, over 10000 trials). Light-blue shading is 90% confidence interval.

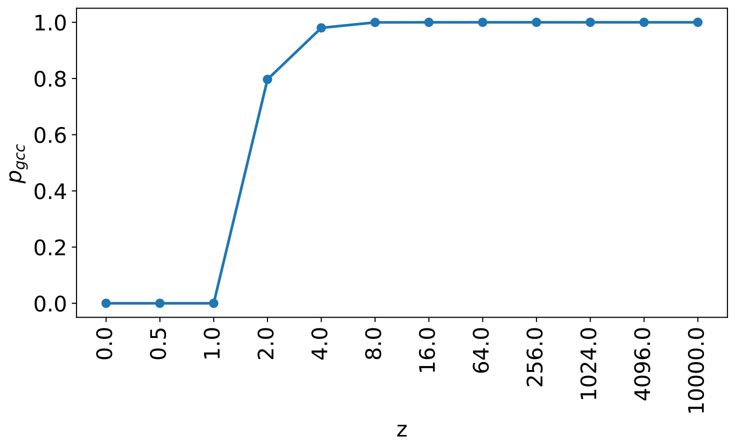

Figure 6. Expected value of p_{gcc} vs. mean degree z. For the LTM having $N = 10000$ nodes, one seed node, and a range of mean degree $z = [0, \frac{1}{2}, 1, 2, 4, 8, 16, 64, 256, 1024, 4096, 10000]$, this shows the emergence of the GCC at mean degree $z = 1$. Thus we need large networks, and a mean degree z large enough to yield a GCC, to have a significant probability of a node belonging to the GCC.

A.4 Individual sample function frequencies and cascade size

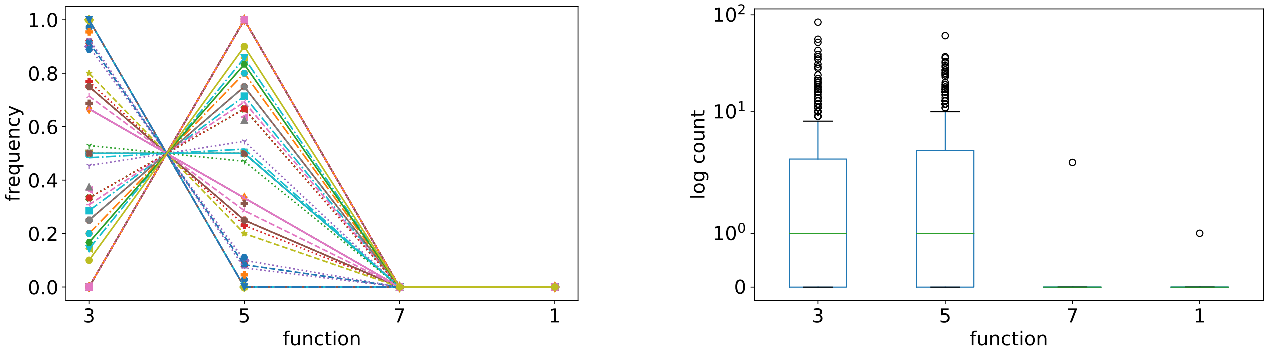

Figure 7. Individual function frequencies correspond to mean values. For all figures, $N = 10000$ over 500 trials. (a) Here we show function distributions for 100 trials having $k = 2$ inputs and fraction of antagonism $\theta = 0$. (Left) The first 100 trials reveal that for each run the functions f_3 and f_5 dominate the frequency, as expected. (Right) A box-and-whisker plot also reveals that individual data follows the expected distribution.

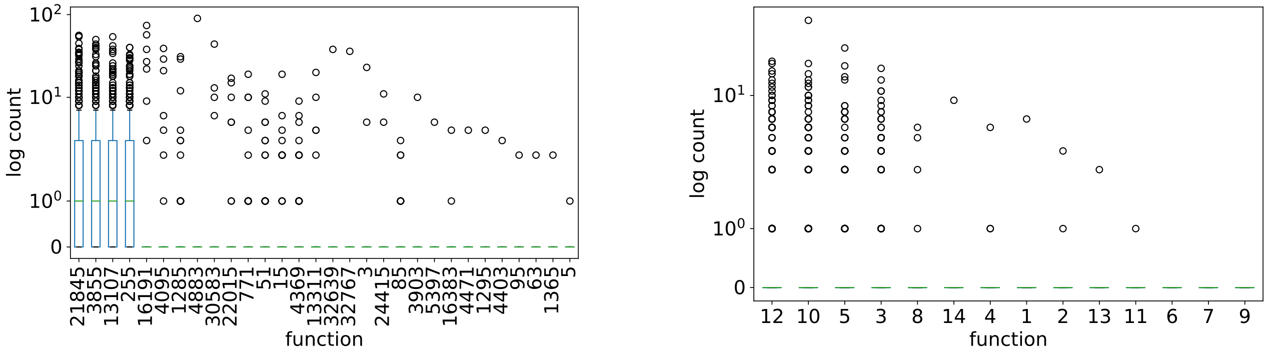

Figure 8. Box-and-whisker plots also reveal that individual data follows the expected distributions, (left) for $k = 4$ inputs and fraction of antagonism $\theta = 0$, (right) for $k = 2$ inputs and fraction of antagonism $\theta = 1/3$.

Here we show how individual trials [Figs. 7, 8] correspond to the ensemble figures appearing in the article [Figs. 3a, 3b, 4b].

References

1. Erdös, P. & Rényi, A. On random graphs i. *Publl. Math. Debrecen* **6**, 290 (1959).

2. Newman, M. *Networks* (Oxford university press, 2018).

3. Novozhilov, A. S. Lecture notes for mathematics of networks (2015).
